# Understanding and evaluating the impact of a multi-institutional academic partnership to reduce cancer health disparities

**DOI:** 10.1186/s12961-026-01496-z

**Published:** 2026-07-17

**Authors:** Ashley Sellers, Meredith Meadows, Dana Marshall, Ashmeet Oberoi, Rebecca Selove, Emilee Ayers, David G. Schlundt, Calandra G. Whitted, Sarah V. Suiter

**Affiliations:** 1 Department of Psychology, Vanderbilt University, 2301 Vanderbilt Place, Nashville, TN 37240 USA; 2 Houston, MS USA; 3 Department of Pathology, Anatomy and Cell Biology, Meharry University School of Medicine, 1005 Dr. DB Todd, Jr. Boulevard, Nashville, TN 37208 USA; 4 Department of Human and Organizational Development, Vanderbilt University, 230 Appleton Place, Peabody #90, Nashville, TN 37212 USA; 5 Center for Prevention Research, Tennessee State University, 3500 John A. Merritt Blvd, Box 9580, Nashville, TN 37209 USA; 6 Meharry-Vanderbilt Alliance, 1005 Dr. D.B. Todd Blvd, Nashville, TN 37208 USA

**Keywords:** Impact evaluation, Multi-institutional partnerships, Collaboration, Workforce development, Cancer health

## Abstract

**Background:**

Cancer health disparities remain a challenge in the USA. To address these disparities, the National Cancer Institute (NCI) launched the Comprehensive Program to Advance Cancer Health (CPACH) in 2001, funding multi-institutional partnerships to strengthen research infrastructure, create training pathways and engage communities. While evaluations of CPACH partnerships often assess single components such as education or community engagement, less is known about their long-term, systemic impact.

**Methods:**

We conducted a qualitative study of the Meharry–Vanderbilt–TSU Cancer Partnership (MVTCP), the longest continuously funded CPACH site. In total, 43 interest holders, including faculty, students, and community advisory board members, participated in semi-structured interviews. Data were analysed using an iterative inductive–deductive approach, guided by the health systems science framework and the Consolidated Framework for Implementation Research.

**Results:**

The analysis revealed four impacts: increased funding/financial ROI, institutional collaboration, educational and career advancement, and community outreach, education and engagement. These impacts were supported by each institution’s capacities, personnel, and collaborative activities, which together generated multilevel change.

**Conclusions:**

The MVTCP demonstrates how multi-institutional partnerships can create synergistic impacts that extend beyond traditional research metrics, producing systemic change in institutions, careers, and communities. Policy and practice should prioritize sustained investment in collaborative models that integrate mentorship and community engagement as structural expectations to improve cancer health for all.

**Supplementary Information:**

The online version contains supplementary material available at 10.1186/s12961-026-01496-z.

## Background

Cancer health disparities are a persistent problem in the USA and require multiple points of intervention to alleviate their impact [1]. Polite and colleagues recommend that efforts to reduce cancer health disparities employ a multipronged approach and identify potential levers of change, including investment in cancer health disparities research across research types (basic, clinical, and population science), advancing community engagement strategies throughout the cancer care continuum and redesigning clinical trials to acknowledge and address cancer health disparities [2]. A through-line in each of these strategies is the need for a well-prepared scientific workforce, as well as a diverse network of institutions with the infrastructure to support expanded cancer research and engagement [2]. These approaches are similar to recommendations made by scholars and policy makers seeking to address other complex population health problems, especially those for which inequality in disease burden is driven by social determinants of health [3–6].

In 2001, the National Cancer Institute (NCI) began its Comprehensive Program to Advance Cancer Health (CPACH) initiative, a funding program intended to reduce cancer burden in the USA by developing cancer research infrastructure at academic and medical institutions that serve underserved populations; creating education and training pathways to increase the cancer research, education, and health care workforce; and improving community involvement and input into cancer research and patient education [7]. Over time, NCI funded 17 CPACH partnerships, each of which involved at least two institutions: an NCI-designated comprehensive cancer center and an institution that serves underserved populations. Endemic to NCI’s theory of change was that the collaborative aspect of the CPACH sites was critical to achieving CPACH goals.

The CPACH partnerships aimed to foster collaboration at multiple levels of human organization, including collaboration among institutions, disciplines, researchers, community members, and scholars at different levels of academic preparation (i.e., students and faculty mentors) [8]. There are multiple known benefits of collaboration in research, service delivery, and multipronged approaches to disease prevention, treatment and recovery. For example, researchers who collaborate across disciplines take advantage of increased knowledge and resources, which often leads to improved performance and innovative output [9, 10]. Researchers who collaborate with community members are better able to translate their research to practice [11] and—when applicable—tend to have an easier time recruiting participants to studies, a well-known challenge for clinical trials involving populations affected by health inequities especially [12, 13]. Similarly, when institutions collaborate, they are able to leverage one another’s resources, benefit from economies of scale and have the possibility of improving research capacity at the individual institutions beyond the reach of the collaboration [14].

At the same time, collaborative partnerships have disadvantages because collaboration of all kinds requires time, resources, and trust to support their development and on-going functioning, and each of these currencies is limited [15]. It is often the case, for example, that participation in one collaborative partnership limits an individual or institution’s ability to participate in others [15]. Similarly, multipronged approaches to addressing complex human problems have the benefit of seeking to create change among a constellation of contributing problems [16]. They also run the risk of defusing resources and energy to such an extent that there are insufficient resources for addressing a specific problem, much less the problems in combination [17].

A growing number of efforts to assess and document the outcomes of CPACH sites exist, however, these efforts typically focus on a single aspect of the multipronged strategy such as education [18–25], workforce development [26–31], community engagement [32–37], cross-institutional collaboration [8, 29, 38, 39] or research infrastructure development [40]. Given the importance of the multicollaboration strategies and the multicomponent approach endorsed by NCI, understanding how CPACH site components work together and the broader impact they have remains an opportunity.

### Description of MVTCP

The Meharry–Vanderbilt–TSU Cancer Partnership (MVTCP) is the CPACH-funded partnership that joins Meharry Medical College (MMC), Vanderbilt Ingram Cancer Center, (VICC) and Tennessee State University (TSU), all located in Nashville, Tennessee. Funded in the first cohort in 2001, the MVTCP successfully completed for funding in four subsequent 5-year cycles (2006–2011, 2011–2016, 2016–2021, 2021–2026) and is the longest continually active CPACH site in the USA. Similar to all CPACH sites, the MVTCP supports four large-scale research projects and two pilot projects each 5-year funding cycle and has multiple “cores” that implement the MVTCP’s mission and support the research projects. These cores include the Research Education Core, the Community Outreach Core, and the Planning and Evaluation Core—cores that are common across all CPACH sites, as well as the Biostatistics Core, the Translational Pathology Core, and the Clinical Trials Core (which later became the Population Research and Clinical Trials Core)—shared resource cores that are specific to the MVTCP’s research needs and institutional expertise.

**Fig. 1:**
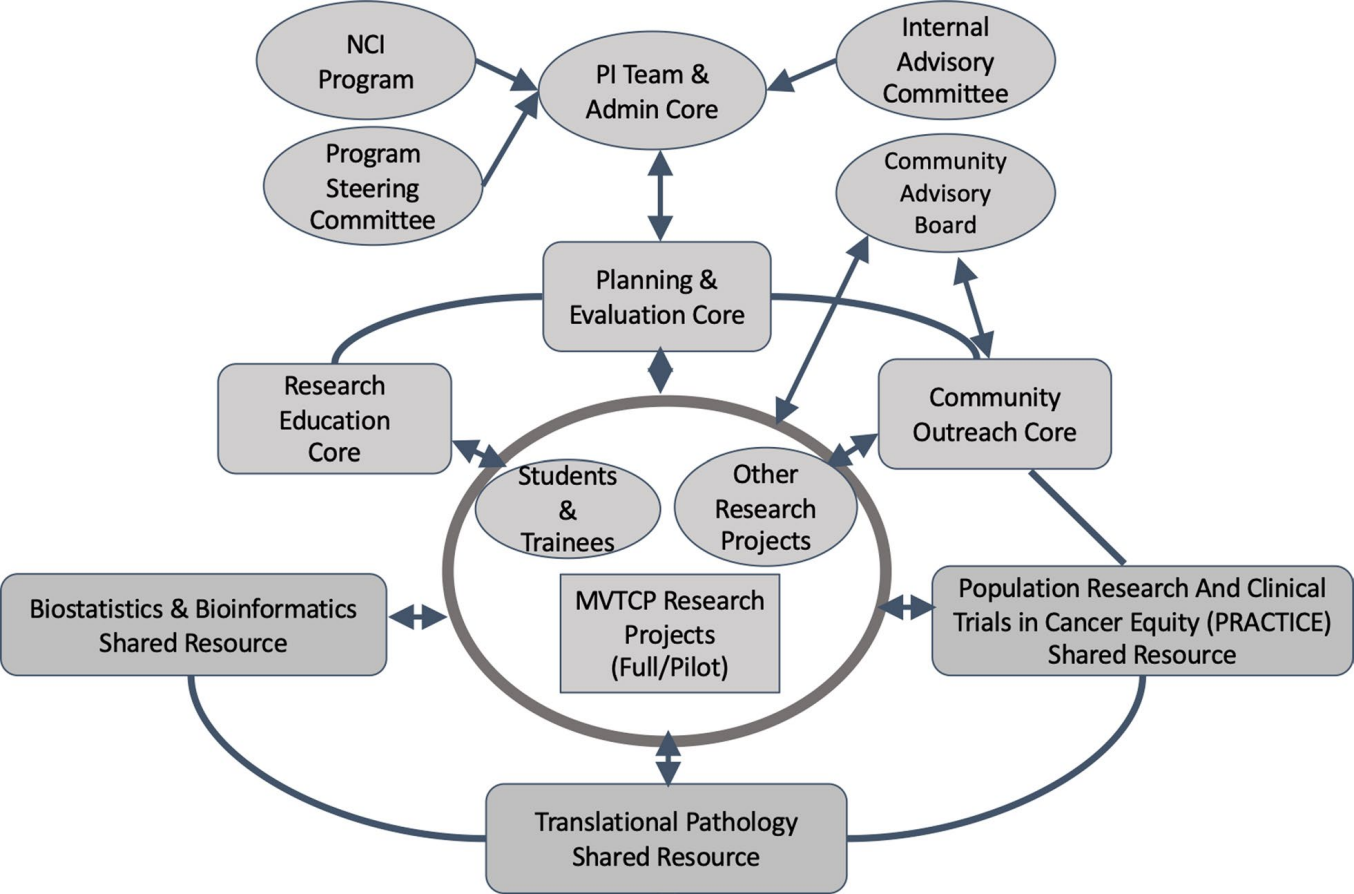
MVTCP partnership diagram

Each research project and core is led by a team of faculty members from at least two of the three institutions represented in the partnership. The MVTCP also has a Community Advisory Board (CAB) that is convened and supported by the Community Outreach Core and provides input on MVTCP research projects, outreach efforts, and programmatic initiatives. The Research Education Core facilitates lab-based cancer research education for students at three high schools in Nashville, undergraduate, and master’s degree students at TSU, medical students at MMC and Vanderbilt University (VU), and PhD students at MMC and TSU (See Fig. 1 for partnership diagram).

The MVTCP Planning and Evaluation Core is tasked with monitoring and assessing the partnership’s processes and outcomes (see the logic model included in the Supplementary Materials) and regularly reports project metrics to stakeholders, including faculty involved in the partnership, project leadership, an external advisory board, and NCI. These project metrics were developed collaboratively by evaluators across the CPACH sites and include 23 items such as number of students participating in research, number of publications authored collaboratively by investigators from different institutions, and number and amount of grant dollars earned as a result of partnership-supported research [7]. As the MVTCP approached its 25th anniversary, leadership and other interest holders expressed interest in better understanding the impact of the MVTCP—beyond standard metrics, how the MVTCP had produced broader sustained change over time.

## Methods

### Study design

Assessing the impact of complex initiatives is challenging [41, 42], and researchers are increasingly turning to qualitative approaches to perform or augment this task [43]. Qualitative approaches excel at capturing context and nuance [44] as well as several of the features known to drive impact (for example, outcome chains, in which one change sets in motion another change, which results in a larger change [45]; or teasing out interventions that contribute to change, even if the intervention was not the sole cause [46]). For this reason, we decided to conduct a qualitative study with broad participant engagement to develop a model of MVTCP’s impact and evaluate the collective efficacy of the partnership in bringing about change. For the purposes of this study, we defined “impact” as changes that are long-term, systems-level, and respond to questions regarding the social, financial, and/or scientific return on investment of the MVTCP. This definition was provided to participants at the outset of the study and the beginning of each interview.

### Participant recruitment

We invited all active faculty members of the partnership, former partnership leaders, and selected students and CAB members who were deemed to be key informants to participate in interviews. Of the 61 people we contacted to invite for an interview, 43 agreed to participate, including 10 students, 3 CAB members, and 30 current and former faculty members. Of the faculty and CAB members, the length of MVTCP involvement ranged from 3 to 25 years, with 11 years being the average length of involvement. Student involvement was somewhat shorter, given that their involvement is linked to the length of their academic programs. The students we interviewed had been involved with the MVTCP between 2–4 years, with an average of 3 years of involvement.

### Data collection

Interviews were conducted between July and October of 2024 via zoom by members of MVTCP Planning and Evaluation Core, which comprised two faculty members and a graduate student from Vanderbilt, a faculty member from Meharry, and a faculty member from TSU, all of whom are co-authors on this paper. When possible, interviewers interviewed faculty from their own institutions or with whom they had previously collaborated, which we believed would provide the most freedom to speak critically of the partnership, especially with respect to collaboration among institutions and resource distribution. All but one of the interviewers conduct qualitative research on a regular basis. The team conducted a practice interview with all evaluation team members prior to beginning data collection. Most interviews lasted between 30–45 min, and all interviews followed a semi-structured interview script (included in the Supplementary Materials). The interview script was designed with questions specific to the MVTCP and aligned with the types of questions recommended by the Most Significant Change technique [47], a strategy developed to assess impact of complex, large-scale initiatives. After the interview, a third member of the evaluation team checked the zoom transcript for accuracy and prepared the transcripts for analysis. All data analysis was completed by the Vanderbilt Qualitative Research Core, a resource that has knowledge of the MVTCP, but no direct involvement.

### Data analysis

Qualitative data coding and analysis was managed by the Vanderbilt University Qualitative Research Core (VU-QRC), a service offered to the MVTCP research community since 2013. Data coding and analysis was guided by the COREQ [48] guidelines, an evidence-based qualitative methodology.

A hierarchical coding system was developed and refined using the interview guide and a preliminary review of the transcripts. Major categories included (1) participant descriptions, (2) research capacity, (3) career development and training, (4) collaboration, (5) impact, (6) project and processes, (7) role of each institution, (8) attitudes and beliefs, (9) barriers and facilitators, (10) strategies for achieving impact, (11) change over time, (12) world events, (13) not observed or experienced and (14) examples. Major categories were further divided from 1 to 10 subcategories, and several categories had additional levels of hierarchal division. Definitions and rules were written for coding categories (coding system included in the Supplementary Materials).

To establish coding reliability, two experienced qualitative coders independently coded two interview transcripts. Coding was then compared, and all discrepancies resolved. After establishing reliability in use of the coding system, they divided and independently coded the remaining transcripts. Each line was treated as a separate quote and was assigned up to 10 different codes. Coded transcripts were combined and sorted by code. Quotations and codes were managed using Microsoft Excel (Office 365) and SPSS version 28.0.

Analysis consisted of interpreting the coded quotes and identifying higher-order themes using an iterative inductive–deductive approach [49]. The goal of the iterative inductive–deductive approach was to develop a conceptual framework that is theoretically informed while integrating resultant content from the qualitative data. Deductively, we were guided by the health systems science framework, as it helps us to understand the interconnectedness of structures, processes, and team science within each institution [50]. We were also guided by the Consolidated Framework for Implementation Research (CFIR), as it guides the identification of strategies to optimize inter-institutional partnerships in achieving shared goals. Inductively, the framework was guided by the qualitative data [51, 52].

## Results

**Fig. 2:**
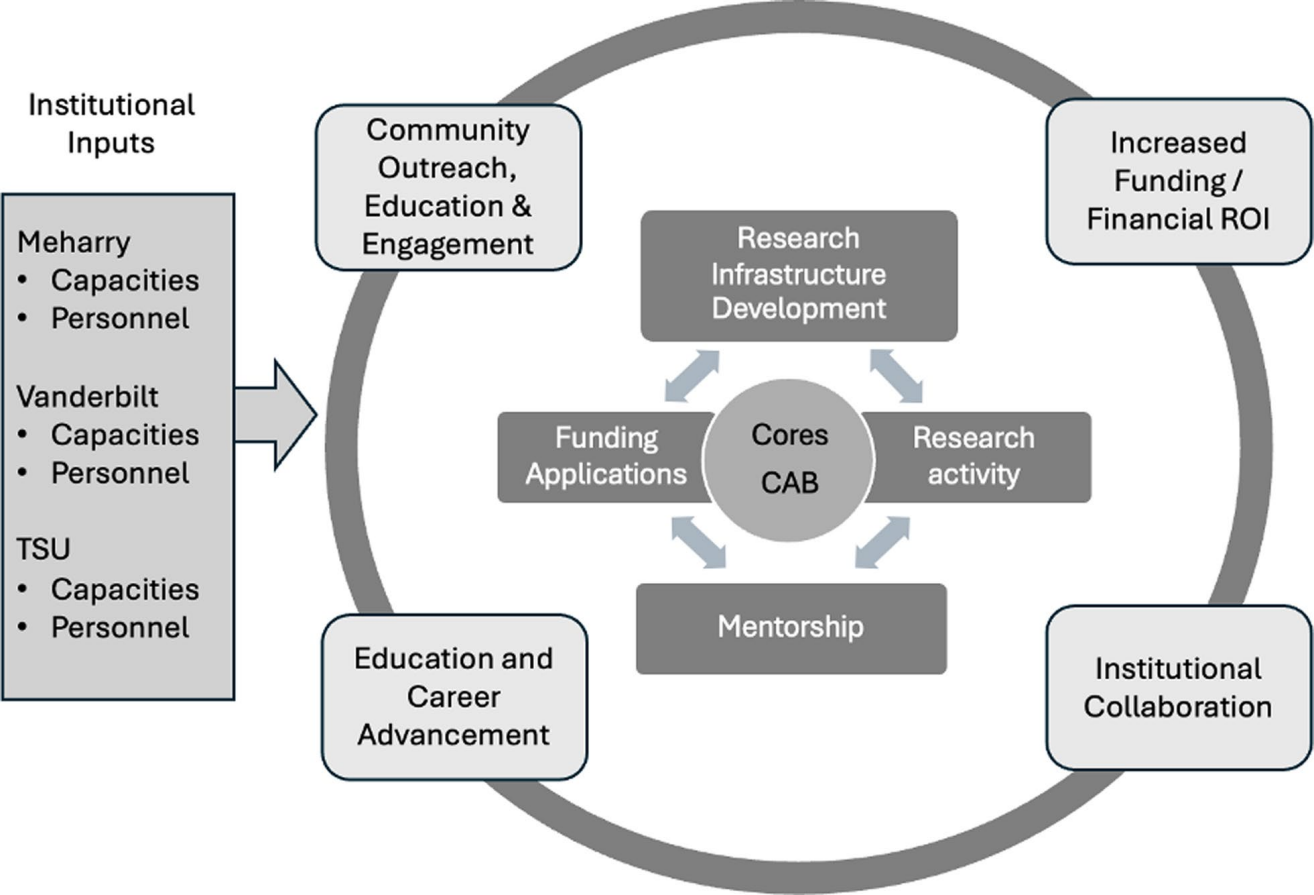
Conceptual framework

The conceptual framework derived from the iterative inductive–deductive analysis is displayed in Fig. 2. This conceptual framework describes the organization, operation, and impacts of the MVTCP on the basis of the qualitative data. On the left side of the figure are the institutional inputs from the three partners: Meharry Medical College, Vanderbilt University and Tennessee State University. Each of these institutions has a unique set of institutional capacities and available personnel. Showing these separately for the three institutions allows for inherent asymmetries among the three institutions to contribute both facilitators and barriers to the partnership.

In the center of the figure, we show three embedded circles. The center circle represents the structure of the MVTCP which is made up of cores. Also included in this inner circle is the Community Advisory Board which interacts with the partnership and the community. Each core has separate personnel with separate and overlapping activities all intended to increase the impact of the partnership. The arrows represent these as elements or components of a dynamic system.

The work done by the partnership includes mentorship, funding applications, research activities, and infrastructure development, which also interact dynamically. The outer circle represents the impacts discussed by participants and includes educational and career advancement, institutional collaboration, increased funding/financial ROI, and community outreach, education and engagement. The remainder of the results will discuss each of these four impacts of the MVTCP, along with the contributing activities for each. Illustrative quotes from participants will be used throughout, and full quotes for each impact are presented in Table 1.

**Table 1:** Illustrative quotes from conceptual framework

Framework theme	Illustrative quotes
Increased funding/Financial ROI	“It is a hard question, but you know I always want to err on the side of ‘yeah.’ You know, I think having an NIH-funded grant looks very, you know, prestigious on your CV. And I think that that allows people to, you know, look directly at that when you’re getting these other awards. So yeah, absolutely. I would say that even though they’re separate in time, having that first grant does set me up or set me apart from other trainees in that regard” (participant 34, Meharry, graduate student). “I needed that financial support that second year, for sure. So that was like incredibly helpful for me in terms of like funding my education and getting that master’s degree. It brought me into contact with people like Dr. [redacted] and Dr. [redacted] – there’s another name over here that’s escaping me at the moment. But also just being connected to those individuals has been really helpful to me in securing some other opportunities or in applying for some further graduate work” (participant 36, TSU, graduate student). “And Vanderbilt has been the same. They provided a necessary infrastructure that was actually the main goal for them to provide for us the infrastructure. So they provided infrastructure, and that we used a lot their of the infrastructure to gain access to more data, and so on, and also of course, the leadership. The partnership was very, very fruitful, at least in my case. The partnership led to many grants that, including the SC1 Grant. That the main grant that I got, at Meharry was due to partnership with [redacted]. My collaboration with [redacted], and I managed to get two DOD-funded grants, which are very difficult to get. As a matter of fact, in 1 year, I was the only one who got one of those idea grants. There were only eight awarded in the whole country. I could not believe, but I think, because of the partnership, ours was funded. So I feel that that was the role that Vanderbilt has played a big big role in helping us establish the right infrastructure for research. And then, of course, now with TSU, they think they have also brought new faculty and new and students to participate” (participant 5, Meharry, researcher).
Institutional collaboration	“So that trust is now there. I can call [redacted] anytime, and we can plan some experiments and can do this without fear of this and that. So that trust to me is one thing that the partnership has really developed between Meharry investigators and Vanderbilt, and even TSU. So there’s that shift. When we have our weekly meetings, you can see that there’s a lot of trust and we speak from the same page. We are on the same page in terms of priorities in terms of goals and foresight. So I think that that is really something that if it were not for the partnership, we still will be in that silos, as far as I’m concerned and people will still be just trying to make things work here” (participant 5, Meharry, researcher). “And then, again, I would say the expertise as well, because I don’t know that we have any other mechanisms for connecting faculty across the institutions without having this partnership in place. Yeah, we reach out to people whenever we have grants that we’re interested in submitting, but oftentimes it’s easier to stay within your own institution, just like, for logistical reasons, even subcontracts are hard to do. So this partnership fosters the development of partnerships. And if you can actually foster productive and meaningful and transdisciplinary where everyone’s bringing expertise, it’s not just a partnership in name, then those collaborations are what our hope is would continue” (participant 14, Vanderbilt, associate director). “I think a lot of it has been more interpersonal in terms of—MVTCP hosts a couple of conferences, for example, and being able to connect with undergraduates and grad students at, like, not only at Vanderbilt, but also at TSU and Meharry. I think that has been really, really just valuable for me. Just because I think seeing their perspectives and seeing like how sort of their views on a lot of just medical education research really just not only helped me make connections with them as potential future colleagues but also just broadens what I see as like the importance of our work” (participant 35, Vanderbilt, medical student). “One idea I think I would suggest is that we need more interactive activities besides just doing research projects. And I think if we have more interactive meetings—we have annual meetings, we have, you know, projects. But I think we can have more regular, maybe monthly meetings, to bring together people. That way they will develop even more projects or develop more collaborations to apply for funding in other places. Cancer Partnership has limited funds, so it can’t fund everyone, but it can, you know, inspire more collaborations among us” (participant 26, Meharry, researcher). “I think definitely like providing a little bit more connection between the institutions, which I know is what we’re trying to work on with the new leaders from each institution, like building that community of practice. Because I know that we have it within each institution, but I think that it definitely could be expanded upon across institutions. Like everybody at TSU knows each other. But I have met maybe a few Vanderbilt students at some of our volunteer opportunities. I don’t think we’ve met any Meharry students. We haven’t gotten to see them very much. So it is one of those things that I know that we’re working on trying to get a little bit more connection, both between students and staff and administrators between each institution. Because I think that’ll even better give us a little bit more connections and things, especially for, like I said, post-bacc career paths” (participant 37, TSU, undergraduate student).
Educational and career advancement	“So since joining the program, I’ve had a lot of opportunities to do research presentations. I’ve done formal ones and informal ones. And I think it’s really helped boost my public speaking ability. At the first, like, symposium that we have for the program, it was my first time doing any sort of research presentation, like a formal research presentation, and I actually won first overall place that year at our symposium. And then, after that, I’ve also done all of the university-wide symposiums for TSU, and I think I’ve placed in almost all of them. And I owe that a lot I feel like to working under [redacted] in the lab. She does a really good job of making sure we know what we’re talking about when we go up there” (participant 37, TSU, undergraduate student).​ “As a component of that, what it’s done is. I mean, I have several students, particularly one that’s coming to mind now, that are in dental school, and, in fact, is about to finish dental school over at Meharry. So for me, the partnership has created a pathway for a lot of the students at TSU that are majoring in biology or chemistry to pursue postbaccalaureate degrees or advanced degrees, whether they’re PhDs and in this case, the professional degree is dentistry. So I think those things would not have happened, because what the partnership did was it gave them exposure access to Meharry as well as it does Vanderbilt” (participant 9, TSU, researcher/mentor/co-PI).​ “It opened my mind to the possibility that I can be a researcher, that that’s something I can do. Because again, I just did not have the confidence or I felt like it was something I couldn’t handle. I think I also was able to gain an understanding that it’s a team effort. So, I worked with [redacted] on creating the manuscript. And then on conducting the interviews, I worked with Meharry’s student graduate assistant. And then when we analysed the data, we worked with [redacted] from Vanderbilt. So, seeing that it’s a team effort and I wouldn’t necessarily have to go through it all by myself and do all of the work, that I can definitely bring in other experts. And one thing [redacted], who was my direct supervisor, mentioned was just the idea of team science. And so bringing in people from different industries that can help to accomplish this one goal of research. And so I liked that we were partnering with our Community Advisory Boards, which were—many were—cancer survivors. And many of them were retired, some of them hold religious leadership, some of them worked in higher education for many years. And so, having their voice on that manuscript as well—to me, I felt like I got to see kind of a team science really play out in the real world and working with them to accomplish that” (participant 43, TSU, graduate student). “So it came out and then provided me the opportunity to build my career as a faculty, do cancer research, prepare and train students, and able to go through the promotion continuum to become tenured full faculty. So that aspect helped me many, many ways” (participant 1, Meharry, researcher).​ “So I was promoted from associate to full professor somewhere along the 3 years. And I have taken on a role of Vice Chair of Health Equity, and also on the VA Side, we recently had a large centre of innovation funded, which is about a 3 million dollar 5 year-grant that I’m in a leadership role for; I’ll be leading our veteran engagement and implementation science core. And so I think all of that was because I was able to leverage a lot of the strengths that I gained through the partnership and the research partnership, to move into those positions” (participant 11, Vanderbilt, researcher). “[redacted], who collaborated so successfully with [redacted] and built strong prostate program at Meharry, and he has been highly successful in getting funding, and he’s a full professor now. And he was a young investigator when he came on. And then there have been others who’ve come on in breast cancer research and different venues. Some stayed, some left. [redacted] was a very strong researcher who Vanderbilt help recruit to Meharry worked on g protein couple of receptors. I collaborated with him we had a DOD grant funding that was joint between Meharry and myself, and he did a phenomenal job. But of course, people who are doing great often get promoted, and he now runs the partnership that’s in [redacted] and is the director of that partnership” (participant 13, Vanderbilt, researcher).
Community outreach, education and engagement	“The other story of that I need to mention is that by teaming up with TSU and forming the community engagement, we gave an opportunity to reach out to the community through churches and other members of the community. But after the partnership right now you turn on the 92.1 which is predominantly African American station, you hear about the Vanderbilt Ingram cancer centre, right. I’ve been on radio a few times. You hear about that. So now we reach the community to the cancer partnership to be able to drive the message, cancer education, cancer prevention and so forth. And that’s why I said during the first 10 years, the number of cases, cancer cases diagnosed in the hospital here which were mostly stage 3, and maybe stage 4 have come down to point where most cases were diagnosed as stage 1 and stage 2, meaning that there were chance for the individuals to go through successful treatment, management and so forth, and survive for a longer time. So those are things that have been very, very important within the community. And then in with TSU they started this program, the HPV vaccine. Right, if you’ve the vaccine and it benefits on young girls as well as even boys, right? Because of the involvement of HPV inherent in cancer and cervical cancer” (participant 1, Meharry, researcher). “My work on the board has been quite extensive. From meeting [redacted], we met monthly, and participating as we listened to cohorts who brought research ideas before the CAB requesting our reflection upon information being presented, sometimes the research concept itself, sometimes asking us to look at documents that will be given to the public. Were those documents relevant? Did they lack sensitivity or inclusivity? And so that was primarily what we have done on that board…” (participant 39, community advisory board member). “The aspects of the partnership that have had the greatest impact has been the community engagement and outreach. It takes time to be able to really see real effects and impact from translational research and basic science. But you can see real immediate impact from community engaged research and community engagement. So I think for me, that’s where the impact has been, and you see it because the stakeholders from the community over the last 11 years or so 12 years of being here, they’ve been participants at several of the annual meetings. So if that community engagement had not been impactful and that outreach had not been impactful, we would not get that kind of participation from the community. That’s probably the strongest impact I’ve seen personally” (participant 9, TSU, researcher). “Then, again, we definitely need to do more to break down barriers to individuals understanding what cancer is, and how to participate in screenings and you know what that means. And I think there’s just a lot of fear around cancer and not really understanding what screenings can do. And so I think that we need to get out of the laboratory and into the community if we really want to address cancer disparities and promote equity and cancer treatment” (participant 12, Vanderbilt, researcher).

### Increased funding/financial ROI

Funding was widely discussed as an impact of the partnership. Participants described how the partnership improved access to financial resources at both the student and faculty level, supporting professional advancement, degree completion, and institutional capacity to secure external grants. Importantly, the partnership’s large federal grant enabled more equitable distribution of funds to MMC and TSU, small institutions that historically have had less access to major research awards.

#### Contributing activities

Participants linked funding impacts to several partnership activities, including financial support for student stipends, structured mentoring that increased exposure to grant-funded research, and the development of research infrastructure that enabled competitive grant submissions.

Students described the partnership as a powerful resource for building credibility and accessing future funding opportunities. One graduate student from MMC noted that “having an NIH-funded grant looks… very prestigious on your CV,” and explained that even when applying for unrelated awards later on “having that first grant does set me apart from other trainees.” In addition to professional advancement, participants emphasized that financial support through the program reduced financial barriers to completing their degrees. A student at TSU reflected that “I needed that support in my second year” and described the funding as “incredibly helpful for me in terms of… getting that master’s degree.”

For faculty, access to shared infrastructure and collaborative relationships across institutions were essential to increasing research capacity leading to grant awards. One researcher at Meharry described how Vanderbilt “provided the infrastructure” that supported access to data and leadership. This infrastructure was seen as important to securing research awards: “The partnership led to many grants… including the SC1 grant [supplemental grant that funds independent research related to the MVTCP] and two DOD [Department of Defense]-funded grants, which are very difficult to get… but I think, because of the partnership, ours was funded.”

### Institutional collaboration

The partnership was recognized for its role in strengthening collaboration across MMC, TSU and VICC. Participants described a shift away from institutional silos toward more aligned goals, improved communication, and improved trust across campuses. These cross-institutional relationships were seen as crucial to building a sustainable research infrastructure and advancing the partnership’s goals.

#### Contributing activities

Participants attributed the shift from institutional silos to collaborative relationships to formal mechanisms introduced by the partnership, such as joint research cores, regular meetings, and mentorship structures that fostered cross-institutional collaboration. One researcher at MMC explained that, prior to the partnership, institutional silos limited cooperation, but “that trust is now there. I can call [colleague at VICC] anytime and we can plan experiments… when we have our weekly meetings, you can see that there’s a lot of trust and we speak from the same page.”

Faculty noted that without the partnership, logistical barriers, such as coordinating subcontracts and identifying collaborators, often made it easier to stay within one’s home institution. A Vanderbilt researcher acknowledged that “we have very few other mechanisms for connecting faculty across the institutions without having this partnership in place.” The partnership cultivated collaborations that were “productive and meaningful and transdisciplinary,” with each institution contributing strengths.

Students also described how cross-institutional events, such as conferences and research programs, helped them engage with peers across campuses. One medical student shared that meeting students from TSU and MMC “was really, really just valuable…seeing their perspectives…not only helped me make connections…but also just broadens what I see as the importance of our work.”

In addition to describing existing collaborative relationships as an impact of the partnership, participants saw the MVTCP as a mechanism for continuing to grow collaboration and made recommendations accordingly. For example, several participants suggested increasing the frequency of interactive activities to strengthen institutional collaboration going forward. One researcher at MMC proposed more monthly meetings, explaining that this could help “develop even more projects… or collaborations to apply for funding in other places.” Students echoed these suggestions and expressed a desire for more networking across institutions. A TSU undergraduate explained that while their intra-institutional networks were strong, they had met only “a few Vanderbilt students” and “haven’t gotten to see [Meharry students] very much,” adding that more connection between students, staff, and administrators “could give us… more career path opportunities.”

### Educational and career advancement

Participants described how the partnership supported career advancement for both students and faculty. For students, the partnership cultivated confidence, research skills, and helped identify future career paths. For faculty, engagement in research and mentorship through the partnership contributed to promotion, visibility, and leadership roles.

#### Contributing activities

Students attributed their career growth to the partnership’s structured mentorship, research experiences, and opportunities to present their work. One undergraduate student from TSU reflected on how repeated presentation opportunities strengthened their scientific communication and self-confidence, explaining “at the first… symposium we have for the program it was my first time doing any sort of research presentation… and I actually won first overall place.” They noted that mentorship from their lab supervisor played an important role in preparing them to share their work effectively.

In addition to skill-building, participants discussed how the partnership created exposure and access to graduate programs. A TSU researcher shared that several of their students have gone on to advanced degree programs because of the institutional connections made possible through the partnership. They explained that “what the partnership did was it gave them exposure access to Meharry as well as…Vanderbilt.”

Graduate students also described how the partnership helped them grow into their identity as researchers, especially through experiences that emphasized team science. One TSU graduate student shared that prior to the program, “I just did not have the confidence or I felt like it was something I couldn’t handle.” Participating in collaborative manuscript writing, data collection, and community-based work shifted their view: “It opened my mind to the possibility that I can be a researcher.” They described seeing “team science really play out in the real world,” with academic and community collaborators all contributing to the research process.

Faculty described parallel benefits. For some, the partnership was described as directly supporting their path to tenure or promotion. A researcher at MMC explained that the program gave them the opportunity to “build my career as a faculty, do cancer research, prepare and train students, and [be] able to go through the promotion continuum to become tenured full faculty.” A Vanderbilt researcher credited the partnership with contributing to their promotion to full professor and leadership roles in both academic and VA-affiliated research centres, stating, “I think all of that was because I was able to leverage a lot of the strengths that I gained through the partnership.”

Several faculty also described how the partnership helped launch the careers of early stage investigators, several of whom went on to lead other cancer research programs. A Vanderbilt participant described one early career researcher who “collaborated so successfully… and built strong prostate program at Meharry, and he has been highly successful in getting funding, and he’s a full professor now. And he was a young investigator when he came on.”

### Community outreach, education and engagement

Participants described community outreach as one of the most visible impacts of the partnership. Through health education, public engagement, and collaboration with local organizations, the partnership was credited with building trust and advancing cancer prevention efforts in historically underserved communities.

#### Contributing activities

Participants emphasized that the partnership’s connections, especially through TSU and Meharry, were essential for reaching communities. One researcher explained that “by teaming up with TSU and forming the community engagement, we gave an opportunity to reach out to the community through churches and other members of the community,” illustrating how trusted community organizations served as access points for outreach.

Community advisory board members also commented on their role in guiding research activities and materials to be responsive to their community’s needs. One board member explained, “my work on the board has been quite extensive… we met monthly… sometimes the research concept itself, sometimes asking us to look at documents that will be given to the public…” This feedback helped refine research protocols and study documents and also ensured that community perspectives were embedded into the implementation of partnership activities.

Media outreach contributed to broadening the partnership’s reach and promoting early cancer detection. A researcher from Meharry noted that “right now you turn on the 92.1 which is predominantly African American station, you hear about the Vanderbilt Ingram Cancer Center… So now we reach the community through the cancer partnership to be able to drive the message, cancer education, cancer prevention and so forth.”

These collective community engagement efforts were seen as building long-term trust. A researcher from TSU shared, “you can see real immediate impact from community engaged research,” noting that over more than a decade, “stakeholders from the community… have been participants at several of the annual meetings.” They added that without meaningful community involvement, “we would not get that kind of participation.” Participants also acknowledged that more could be done. Several felt that not all researchers were equally engaged in outreach and emphasized the need for even more interaction with community members. “We need to get out of the laboratory and into the community if we really want to address cancer disparities,” one Vanderbilt researcher explained, adding that there is still “a lot of fear around cancer” and confusion about what screenings mean.

## Discussion

This study explored the long-term, multilevel impact of the Meharry–Vanderbilt–TSU Cancer Partnership (MVTCP), the longest continuously funded CPACH site in the USA. While most evaluations of CPACH initiatives have focused on a single component [8, 18–40], our findings show how these elements interact to create systemic impact. More broadly, there are several calls for multi-institutional and multilevel approaches to complex health challenges in the academic literature, as well as conceptual frameworks for implementing these approaches [3–6]; however, there are few studies that evaluate the impact of these approaches [53]. For example, a recent scoping review of institutional partnerships aimed at reducing health disparities by promoting diversity in the academic health sciences found that existing literature (which was scant) primarily described steps for developing partnerships (identifying partners, engaging community members, outlining program goals, obtaining funding) and process evaluation of implementation goals. The studies that reported outcomes measured them in focused domains (for example, career outcomes) as the CPACH studies did, and there was little-to-no impact measurement [53]. The study and reporting of focused outcomes in particular domains is important, and literature to which we (the authors of the present study) have contributed. Nevertheless, our study of the longer-term and systems-level impacts of the MVTCP makes an important contribution to existing literature. Themes from the qualitative analysis demonstrate that the partnership contributed to synergistic, multilevel change by aligning resources, institutions, and communities around a common goal of reducing cancer health disparities. The conceptual framework derived from stakeholder interviews illustrates how MVTCP functions as a model of team science, bringing together students, faculty, and community members to advance research, education, and engagement. These results point to the importance of multi-institutional partnerships in addressing complex health disparities and offer lessons for replication in other areas of population health research.

### Multilevel financial outcomes

Participants described the financial impact of the partnership, including distribution of funding to smaller, less historically resourced institutions, stipends for students, and infrastructure development that enabled faculty to secure external awards. While grant mechanisms tend to concentrate funding within already well-resourced institutions [54], MVTCP enabled smaller institutions to access infrastructure and mentorship that facilitated their ability to compete for awards. This redistribution shows how funding mechanisms can act as levers for equity in research and generate cascading impacts across multiple levels of a partnership. Importantly, these findings also show the policy relevance of grant mechanisms that incentivize multi-institutional collaboration rather than concentrating resources.

### Collaboration, community engagement and structural change

The MVTCP fostered a cultural and structural shift away from institutional silos toward cross-institutional collaboration, trust, and shared goals. Collaboration in this context extended beyond individual relationships to reshape organizational norms. Participants noted that formal mechanisms (such as research cores, regular meetings, and joint projects) created sustainable inter-institutional ties. These changes advanced scientific output and reshaped organizational structures to sustain collaboration beyond individual projects.

Collaboration was not confined to academic institutions. The partnership’s community advisory board and relationships with community organizations aimed to embed community engagement into the collaborative structure. In this way, community engagement functioned as both an outcome and a collaborative activity: it strengthened trust among historically underserved populations, supported recruitment into research, and guided institutional activities to community priorities.

These findings reinforce health systems science perspectives that structural supports are necessary for sustaining collaborative work across organizations [15]. The partnership can also serve as an illustration of how strategic design can embed team science principles into the structure of institutions [55], creating models that may be transferable to other areas of health disparities research.

### Career pipeline

The partnership’s multilevel training format was viewed as a defining feature of its impact. Notably, the training pipeline shows how investments in mentorship can build a diverse workforce positioned to address cancer disparities. Students described increased confidence, skills in scientific communication, and exposure to academic careers. Faculty credited the partnership with supporting tenure and promotion, as well as launching the careers of early stage investigators who now lead independent research programs. In the context of workforce shortages in oncology practice and research [56, 57], this model demonstrates how multi-institutional partnerships can expose students to careers in cancer research and expand participation of historically underrepresented groups to create long-term returns on federal investment.

### Practical implications

The findings suggest that sustained, funded multi-institutional partnerships can create synergistic impacts that extend beyond traditional research metrics. For policy and practice, this suggests the need to incentivize multi-institutional models that distribute resources effectively and strengthen smaller institutions. These synergistic impacts also point to the need to invest in long-term infrastructure, including shared research cores and evaluation systems, to ensure sustainability. Although certain aspects of this partnership could likely be sustained without continued funding, it is nevertheless the case that the CPACH funding mechanism provides resources and incentives for the challenging but impactful work of addressing cancer health disparities through multidimensional strategies. Disruptions in funding could negatively impact the development of this infrastructure.

In addition, structured mentorship pipelines are needed to prepare the next generation of researchers, and community engagement should be embedded as a structural expectation rather than an optional activity. Institutions must prioritize communication systems to maintain this mentorship and engagement and increase collective impact. These lessons emphasize that multi-institutional collaborations can play a unique role in addressing cancer health disparities if designed and supported with equity and longevity at their core.

### Limitations and future directions

Although findings illustrate systemic impacts, they are based on perspectives from a single partnership; therefore, generalizability may be limited. While MVTCP is a long-standing partnership and is thus uniquely positioned to illustrate sustained change, its specific institutional and community context may limit transferability to other partnerships. The study also relied on retrospective reflections from faculty, students, and community members; as with any long-term initiative, recall bias may shape how events and outcomes are described.

Future research should build on these findings by comparing outcomes across multiple CPACH partnership to identify common structural features that facilitate or constrain impact. Longitudinal and embedded qualitative designs could further illustrate how partnerships evolve across successive funding cycles and how stakeholders sustain impact. Additionally, applying this framework to other areas of health disparity research would help assess the transferability of multi-institutional partnership models and refine strategies for sustaining collaborations.

## Conclusions

This study demonstrates that the impact of MVTCP extends beyond individual projects or short-term outcomes. By redistributing resources, expanding collaboration across institutions, facilitating a career advancement pipeline and embedding community engagement, the partnership has produced synergistic change across multiple levels of cancer research. These findings affirm that addressing cancer health disparities requires durable, multi-institutional structures rather than isolated interventions. As CPACH partnerships approach their third decade, the lessons from MVTCP bring to light the importance of sustained investment and intentional design in ensuring that these collaborations endure and continue to evolve in ways that meaningfully improve cancer outcomes.

## Supplementary Information

Supplementary Material 1.

Supplementary Material 2.

Supplementary Material 3.

## Data Availability

The datasets generated and analysed during the current study are not publicly available owing to the in-depth descriptive nature of the qualitative data and the risk of identifying participants or the people about whom they are talking; however, data are available from the corresponding author upon reasonable request.

## Abbreviations

CAB: Community advisory board
CFIR: Consolidated Framework for Implementation Research
CPACH: Comprehensive Program to Advance Cancer Health
MMC: Meharry Medical College
MVTCP: Meharry–Vanderbilt–TSU Cancer Partnership
NCI: National Cancer Institute
TSU: Tennessee State University
VICC: Vanderbilt Ingram Cancer Center
VU-QRC: Vanderbilt University Qualitative Research Core
