# Supplementary material for: Understanding and evaluating the impact of a multi-institutional academic partnership to reduce cancer health disparities
Source: Health Res Policy Syst. 2026 Jul 17;24:59. doi: 10.1186/s12961-026-01496-z (PMC13377699; doi:10.1186/s12961-026-01496-z)
Supplement: Supplementary file 1 — Supplementary Material 1. [file 12961_2026_1496_MOESM1_ESM.docx]

**Questions to guide MVTCP Impact Study – Faculty Protocol**

Thank you for agreeing to speak with me today. I am an evaluator working on assessing the impacts of the MVTCP. We are speaking to you today because you were identified as a key informant who can provide your perspective on the impacts of MVTCP. While impact can mean different things to people, we are most interested in understanding impact as the most significant changes that have occurred over time as result of the Partnership. Our conversation today will help inform the evaluation of MVTCP and the reporting of our impacts to the NIH. There are no right or wrong answers.

We plan to use Zoom’s auto-transcription feature to transcribe your interview. After the interview is over, we will check the transcript for completeness and accuracy. We will also de-identify the transcripts after each interview, although in some cases (e.g., when interviewing long-standing PIs of the partnership), it will be difficult to fully conceal identities. We will store the transcripts in a password protected file, and only members of the evaluation team will have access to them. Do you have any questions before we begin?

1. Please briefly describe your role/position at your institution?
2. Describe your how your project team came together

**Probe:** How did you become involved in the MVTCP?

**Probe:** How did you find one another?

1. From your perspective, what are the roles and contributions of each of the 3 institutions to the partnership?
2. Are there things that have been accomplished as a result of your project that could or would not have happened without it?

**Probe:** You might think about things that have happened for you professionally, for students, at your institution, or in your community

**Probe:** How has the MVTCP contributed to these changes?

1. From your perspective, what is the most significant impact (long-term change) of the MVTCP on your work?

**Probe:** What specific situations, tasks, actions, and/or results can you relate to this change?

1. One of the goals of the MVTCP is reducing cancer health disparities and/or promoting cancer health equity. When you think of the impacts you described above, are there ways in which they have addressed cancer health disparities or health equity? If so, how?
2. If the NCI were to ask you, “Why should we keep funding this partnership?” what would you say?
3. Going forward, are there impacts you would like to see the MVTCP generate that it hasn’t yet?

**Questions to guide MVTCP Impact Study – Student Protocol**

Thank you for agreeing to speak with me today. I am an evaluator working on assessing the impacts of the Meharry-Vanderbilt-TSU Cancer Partnership (MVTCP). We are speaking to you today because you were identified as a key informant who can provide your perspective on the impacts of MVTCP. While impact can mean different things to people, we are most interested in understanding impact as the most significant changes that have occurred over time as result of the Partnership. Our conversation today will help inform the evaluation of MVTCP and the reporting of our impacts to the NIH. There are no right or wrong answers. Do you have any questions before we begin?

1. Tell me a little bit about your experience and involvement with the MVTCP. What did you do while you were involved with the MVTCP?

- How long were you involved?
- How did you first get connected or involved?
- I know you were a student at _____ while you were involved with the partnership. Were you involved with people from any other institution (research mentor, student collaborators, lab experience, etc.)?

1. What were some of your accomplishments while you were involved with the MVTCP? These can be formal accomplishments (e.g., publishing a paper) or informal (e.g., gaining confidence, learning new things)?
2. Was there anything you were able to experience or accomplish during your involvement with the MVTCP that you wouldn’t have had the opportunity to do otherwise?
3. Did your experience with the MVTCP affect your educational or career goals? If so, how?
4. The MVTCP is a research training program, among other things. What impact, if any, has the MVTCP had on your own research or views of research that we haven’t already talked about?
5. From your perspective, what is the most significant impact of the MVTCP on students?
6. One of the goals of the MVTCP is to reduce cancer health disparities and/or promote cancer health equity. When you think of the impacts you described above, are there ways in which they relate to cancer health disparities and/or health equity? If so, how?
7. What are some of the things the MVTCP could do better or differently to have more of an impact on students?

**Questions to guide MVTCP Impact Study – CAB Member Protocol**

Thank you for agreeing to speak with me today. I am an evaluator working on assessing the impacts of the MVTCP. We are speaking to you today because you were identified as a key informant who can provide your perspective on the impacts of MVTCP. While impact can mean different things to people, we are most interested in understanding impact as the most significant changes that have occurred over time as result of the Partnership. Our conversation today will help inform the evaluation of MVTCP and the reporting of our impacts to the NIH. There are no right or wrong answers.

We plan to use Zoom’s auto-transcription feature to transcribe your interview. After the interview is over, we will check the transcript for completeness and accuracy. We will also de-identify the transcripts after each interview, although in some cases (e.g., when interviewing long-standing PIs of the partnership), it will be difficult to fully conceal identities. We will store the transcripts in a password protected file, and only members of the evaluation team will have access to them. Do you have any questions before we begin?

1. Please briefly describe your role/position within the MVTCP? What are your primary responsibilities as a CAB member?
2. How did you become involved in the MVTCP?
3. From your perspective, what are the roles and contributions of the CAB to the partnership?
4. Are there things that have been accomplished as a result of the CAB that could or would not have happened without it?

**Probe:** You might think about things that have happened for you professionally, for students, at your institution, or in your community

**Probe:** How has the MVTCP contributed to these changes?

1. From your perspective, what is the most significant impact (long-term change) of the MVTCP on you and/or the community you represent?

**Probe:** What specific situations, tasks, actions, and/or results can you relate to this change?

1. One of the goals of the MVTCP is to promote cancer health for all. When you think of the impacts you described above, are there ways in which they have contributed to this mission? If so, how?
2. If the NCI were to ask you, “Why should we keep funding this partnership?” what would you say?
3. Going forward, are there impacts you would like to see the MVTCP generate that it hasn’t yet?
