# Supplementary material for: Understanding and evaluating the impact of a multi-institutional academic partnership to reduce cancer health disparities
Source: Health Res Policy Syst. 2026 Jul 17;24:59. doi: 10.1186/s12961-026-01496-z (PMC13377699; doi:10.1186/s12961-026-01496-z)
Supplement: Supplementary file 2 — Supplementary Material 2. [file 12961_2026_1496_MOESM2_ESM.pdf]

MVTCP Impact Study Coding Framework

Code	Label	Definition	Notes/Rules
1	**Stakeholder description**	**Discussion on the stakeholder's background**	
1.1	Location	Discussion on stakeholder's location (e.g. MMC, VICC, TSU)	**Designated column**
1.2	Institutional role	Discussion on the stakeholder's role (e.g., faculty member, professor, etc.) and responsibilities	Do not code for cancer partnership roles
1.3	Partnership role	Describes his/her role (e.g., PI, epidemiologist, etc.) and responsibilities in the cancer partnership	**Designated column**
1.4	Learned about partnership	Discussion on how they learned about the parnership	
1.5	Involvement years	Discussion on how long the participant has been involved in the partnership	
1.6	Prior roles	Discussion on stakeholer's prior roles	
1.7	Other background for stakeholder	Other stakeholder background not mentioned above	
2	**Research Capacity**	**Apply this code when participants refer to the capacity to conduct research including research infrastructure, training, and grants.**	
2.1	Infrastructure Development	Apply this code when participants refer to the development of physical research infrastructure including the use of labs.	Expanding physical space, etc.
2.2	Institutional Development	Apply this code when participants refer to the development of research capacity at the institutional level such as executive office and administrative support for research.	Higher level admin support
2.3	Department and Center Development	Apply this code when participants refer to the development of research capacity at the department or center level including recruiting new faculty, and building out a center.	Linking research groups together, building out recruitment strategy, etc.
2.4	Grant applications	Apply this code when participants refer to competitive grants including applying, receiving, or being rejected from grants.	
2.4.1	Competitiveness	Discussion on how he/she did not receive funding for research but a different lab/researcher obtained funding for the same/similar projects	Also code if the participant discusses having to apply multiple times
2.4.2	Applying for funding	Discussion on how the researcher applied for funding, including the steps that they took, etc.	
2.4.3	Funding status	Discussion on the status of funding (e.g., received or rejected)	
2.4.4	Other funding methods	Discussion centers on other funding methods that were used	Other methods outside of grant funding
2.5	Other research capacity	Other research capacity not otherwise captured	
3	**Career Development & Training (CD&T)**	**Apply this code when participants refer to career development and training of anyone involved in the partnership.**	
3.1	CD&T of Students	Apply this code when participants refer to the career development and training of high school, undergraduate, and graduate students specifically.	
3.2	CD&T of Post-docs	Apply this code when participants refer to the career development and training of post-doctoral fellows.	
3.3	CD&T of Early-career faculty	Apply this code when participants refer to the career development and training of early-career faculty.	
3.4	Other CD&T	Other reference to CD&T not mentioned above	
4	**Collaboration**	**Apply this code when participants refer to collaboration in the partnership.**	
4.1	Partnership Building	Apply this code when participants refer to high-level collaborations between either Meharry, Vanderbilt, and TSU as institutions such as MVTCP and other cross-cutting intiatives.	
4.2	Research Collaboration	Apply this code when participants refer to collaborations in research development and activities between researchers at either Vanderbilt, Meharry, and TSU.	Do not code if the participant discusses the role that each institution plays in the partnership
4.3	Continuing collaboration	Discussion on how collaboration continues between researchers after one leaves the organization	
4.4	Lapse in collaboration	Discussion on a timeframe where there was no partner to collaborate with	
4.5	Other collaboration	Other collaboration not discussed above	
5	**Impact**	**Apply this code when participants refer to current and potential impacts of the researcher or partnership**	
5.1	Partnership Impact	Apply this code when participants refer to impacts achieved by the partnership.	
5.2	Lack of Impact	Apply this code when participants refer to desired impact that has not been achieved.	
5.3	Other impact	Other impact not mentioned above	
6	**Project and Processes**	**Discussion centers on the project specific tasks**	
6.1	Project focus	Participant discusses the focus of the project/research	
6.2	Project process	Participant discusses parts of the project's process (i.e., how patients were recruited)	
6.3	Other project and process	Other project and process not otherwise mentioned	
7	**Role of each institution**	**Participant's perception of the role of each institution for the cancer parternship**	
7.1	Role at Vanderbilt	Perception of Vanderbilt's role	
7.2	Role at Meharry	Perception of Meharry's role	
7.3	Role at TSU	Perception of TSU's role	
8	**Attitudes and Beliefs**	**Attitudes and Beliefs specific to the participant**	
8.1	Normative beliefs	Discussion centers on participant's perception and expectation of others' beliefs	
8.1.1	Injunctive normative beliefs	Participant perception of what is expected of themselves. Also includes participants own expectation for others' behavior.	
8.1.2	Descriptive normative beliefs	Participant perception of the behavior of those around her/him. Perception of what others actually do.	
8.2	Concerns	Discussion mentions concerns	
8.3	Comparison	Any comparison made	
8.4	Behavioral beliefs/attributions	Discussion centers on causal attributions related to a given situation or condition	
8.5	Self efficacy/control beliefs	Discussion centers on patient's belief in her/his own capacity, capability, and control to engage/execute a given task	
8.6	Self perception	Way that participant views her/himself (e.g., personality, dispositional traits)	
8.7	Attitudes toward the institutional partnership	Attitudes toward the cancer partnership between Meharry, Vanderbilt, and TSU	
8.8	Value placed on research	Attitudes toward and value of research	

8.9	Attitudes toward funding	Participant's attitudes toward funding	
8.10	Other attitude and belief	Other attitude and belief not mentioned above.	
**9**	**Barriers and facilitators**	**Apply this code when participants refer to barriers and/or facilitators in executing the mission and activities of MVTCP and identify hurdles in improvement for the partnership or in achieving impact.**	
9.1	Barrier/challenge	Discussion centers around a barrier or disadvantage	
9.2	Facilitator/advantage	Discussion centers around a facilitator or advantage	
9.3	Contingency/conditional	A decision or choice depends upon specified factors	
**10**	**Strategies for Achieving Impact**	**Apply this code when participants refer to ideas to help the partnership to achieve its goals.**	
**11**	**Change over time**	**Any discussion centering on change over time**	
**12**	**World events**	**Events such as Tuskegee, COVID-19, etc.**	
**13**	**Not observed/experienced**	**Participant has not observed or experienced a given situation**	
**14**	**Examples**	**Participant provides any example (i.e., personal, peer, hypothetical)**	
	HE-Focused Research at Cancer Center	Apply this code when participants refer to research focused on health disparities at Vanderbilt.	Sub-topical
	HE-Focused Research at ISUPS	Apply this code when participants refer to research focused on health disparities at Meharry or TSU.	Sub-topical
	HE-Focused Education and Curriculum	Apply this code when participants refer to education or training focused on health disparities.	
	Requirements	Participant discusses the requirements of the study (e.g., sample type and number of samples)	
	Community Outreach	Apply this code when participants refer to community outreach in the partnership.	Topical
	Engagement with Public	Apply this code when participants refer to engagement with the broader community such as in clinics and through educational outreach events.	Sub-topical
	Community Advisory Board	Apply this code when participants refer to the Community Advisory Board.	Sub-topical
	Knowledge	Participant discusses their knowledge level of the research and partnership	
	Knowledge of role	Discussion on the knowledge of the participant's roles and responsibilities in their job role	
	Researcher knowledge	Discussion on the knowledge of research and different role's responsibilities	
	Partnership goal	Participant disucsses the goal of the cancer partnership	
