# Supplementary material for: Understanding and evaluating the impact of a multi-institutional academic partnership to reduce cancer health disparities
Source: Health Res Policy Syst. 2026 Jul 17;24:59. doi: 10.1186/s12961-026-01496-z (PMC13377699; doi:10.1186/s12961-026-01496-z)
Supplement: Supplementary file 3 — Supplementary Material 3. [file 12961_2026_1496_MOESM3_ESM.docx]

| **High-level Logic Model of MVTCP from 2021-2026 Funding Cycle** | | | | |
| --- | --- | --- | --- | --- |
| **Activities** |  | **Outputs** |  | **Outcomes** |
| **Administrative Core** | | | | |
| - Strategic planning & priority setting - Provide and maintain infrastructure for implementation & Integration of all MVTCP entities - Selection and development of research projects - Review & assess progress of MVTCP cores & projects - Identify & leverage internal & external resources to further goals of MVTCP - Convene, coordinate, & communicate w/ IAC & PSC - Communicate with NCI & respond to requests - Plan & implement Annual Symposium - Identify, recruit, mentor, and resource ESIs conducting CHD research |  | - Evidence of strategic plan with clear priorities - Existence of coordinated & functioning infrastructure for MVTCP - # of research proposals, projects selected - Core reviews and projects conducted on an on-going basis - #, type, and institution of resources leveraged - # of PI, IAC, and PSC meetings; attendance at meetings - # & timeliness of responses to NCI requests - #, type, and institution of Symposium attendees - # of ESIs recruited and mentored |  | - MVTCP is driving innovation in education, research & treatment to reduce CHD - MVTCP projects & cores are successful in accomplishing proposed outcomes & milestones - Unfunded projects are competitive in the future - ↑ in resources leveraged to support MVTCP efforts - IAC and PSC advice is garnered and heeded - MVTCP participates fully in NCI PACHE program - ↑ of CHD among MVTCP partners, students, and community, ↑ connections among partners, students & community - ↑ # of ESIs successfully recruited, promoted/tenured, and retained - ↑ # of ESIs awarded independent external grants |
| **Research Projects** | | | | |
| - Identification of research collaborators - Study design & implementation - Data collection, storage, and analysis - Dissemination of findings - Apply for external funding - Leverage core resources as needed |  | - #, qualifications, and institutions of collaborators - Study is designed and implemented according to project plan & milestones - Data are collected, stored, analyzed - # of publications, abstracts & grants submitted - Use of core resources |  | - # of publications - # of abstracts - # of conference presentations - # and type of externally funded grants - Career advancement of project investigators |
| **Research Education Core** | | | | |
| For HS & UG students:   - Summer and academic year research - Cancer research course - Mtgs w/ UG students/ COP - Alumni Affiliate (CRAE) for HS students - Near-peer mentoring with MPH, MD & PhD students / participation in COP for UG students   For URM MPH, MD, & PhD students   - Identification & recruitment to program - Mentor pairing - Cancer research experience - Career development & training - Opportunities to mentor HS & UG students |  | - # of HS students participating in:   - - Summer & academic yr. research     - Cancer course     - COP & CRAE - # of UG students participating in:   - - Summer & academic year research     - Forum & Entering Research Courses     - Near-peer mentoring - # of mentors for HS, UG, MPH, MD, & PhD students - # of students by institution, degree, & year - Participation in activities and opportunities (e.g. mentoring HS & UG students) - # of MD students mentoring TSU students - # of PhD trainees applying for fellowships (e.g. F31) |  | - ↑ participation of URM students in cancer research - ↑ # of URM students matriculating to next level of cancer research education - ↑ sense of science identity - ↑ sense of community - Improved faculty mentoring - Improved institutional resources to support cancer research opportunities for HS & UG students - ↑ participation of URM students in cancer research - ↑ # of URM students pursuing & successful in a cancer research career, oncology careers and/or interested in cancer health disparities - ↑# of minority scientists in federal, private, and academic research sectors - Improved professional skills & confidence |
| **Community Outreach Core** | | | | |
| - Maintain active CAB - Expand community engagement - Assess community needs - Communication among patients, community, researchers - Meet with research project teams - Technical assistance - Research studios - Develop modules, materials & web-based tools - Mentorship and capacity building in CeNR - Build community capacity for research |  | - # and demographics of CAB members - # of meetings; attendance at meetings - Community members full partners on research teams - Documented CAB and COE input into existing projects and dissemination efforts - Documented CAB & COE input into new projects applications & study/study materials design - # and type of resources created, # of website hits, # of social media shared, etc. - # and type of participants engaging with CER trainings; # of trainings held |  | - ↑ alignment among MVTCP research, clinical efforts and identified community needs - ↑ grant success - ↑ study enrollment and retention - ↑ dissemination of findings to community - ↑ awareness & knowledge of cancer prevention and control - ↑ student, faculty, and community capacity for research |
| **PRACTICE Core** | | | | |
| - Develop and maintain collaboration between NGH CTO and VICC CTO - Provide access to existing VICC resources to patients at NGH - Bring new studies to NGH - Leverage NGH resources in facilitating access to supported trials - Develop and maintain centralized resource - Leverage MMC SRU resources |  | - Regular meetings between NGH CTO & VICC CTO leadership - # and demographics of NGH patients accessing VICC resources - # of current & new interventional & observational studies at NGH - # of patients eligible, accrued, & completing - # # & demographics of patients participating in PRACTICE core clinical & population science studies |  | - Strong partnership b/t NGH CTO & VICC CTO - ↑ access to VICC resources for patients at NGH - ↑ awareness, trust and willingness among NGH patients to participate in clinical trials - ↑ participation of NGH patients in clinical trials - Improved clinical trials infrastructure at NGH - ↑ # and diversity of people participating in population science studies |
| **Translational Pathology Core** | | | | |
| - Vet applications for use of resource - Tissue collection & storage - Liquid biopsy - Staining & histology - Microscopy - Pathology - Development & implementation of new methods & services for MMC & TSU investigators |  | - # of tissue samples by: - Total - Organ - Race/ethnicity - SES - # of cancer related microscope hours - # of analyses |  | - Increase access to tissues from diverse populations   - # of labs using resource   - # of investigators using resource - Improve cancer-related pathology services for patients served by Metro General Hospital - Improve cancer research infrastructure for MVTCP |
| **Biostatistics Core** | | | | |
| - Research Education: Office hours at Meharry, Journal club, Seminars, Summer institute - Research Consultation: Hypothesis formulation, Proposal development, Methods recommendations, Analysis, & Write-up - Research Participation & Support: Database development, Develop new methods, Analyze data, Interpret results |  | - # of education activities - # of people participating in education activities - # of studies supported - #, type, and institution of investigators supported |  | - ↑ in MVTCP investigators’ knowledge of & access to biostatistical methods & services - Improved quality of submitted grant proposals - Published papers demonstrate scientific innovation and impact in areas of: - Basic science - Clinical use - Public health use |
| **MVTCP-Wide / Common Activities Across Cores** | | | | |
| - Develop collaborative research projects focused on cancer health equity that involve MMC, VICC, and TSU investigators - Collaborate with other MVTCP cores and projects - Education and mentorship of students, trainees, and junior faculty in health disparities research - Facilitating connections between prospective grantees and research experts - Provide grant consultations |  | - # of collaborative research projects - # of new MMC, VICC, & TSU investigators in collaborative projects - # & type of collaborations with MVTCP cores & projects - #, institution & demographics of students, trainees, and faculty - # of research experts recruited to review studies - # of grant consultations |  | - ↑ number of collaborative research projects among MMC, VICC, and TSU investigators - ↑ grant funding for & publications from collaborative research projects - ↑collaboration with projects & cores - ↑ knowledge of cancer health disparities research among students, trainees, and faculty - ↑ #, types, and success of grant submissions on grants with MVTCP Core support - Increase CHD scholarship: # of published papers, # of abstracts, # of invited presentations by students, trainees, and faculty |
